# Crystal structure of ethyl 2-(2-{(1*E*)-[(*E*)-2-(2-hy­droxy­benzyl­idene)hydrazin-1-yl­idene]meth­yl}phen­oxy)acetate

**DOI:** 10.1107/S2056989014027273

**Published:** 2015-01-03

**Authors:** Mehmet Akkurt, Joel T. Mague, Shaaban K. Mohamed, Eman A. Ahmed, Mustafa R. Albayati

**Affiliations:** a Department of Physics, Faculty of Sciences, Erciyes University, 38039 Kayseri, Turkey; b Department of Chemistry, Tulane University, New Orleans, LA 70118, USA; c Chemistry and Environmental Division, Manchester Metropolitan University, Manchester M1 5GD, England; d Chemistry Department, Faculty of Science, Minia University, 61519 El-Minia, Egypt; e Chemistry Department, Faculty of Science, Sohag University, 82524 Sohag, Egypt; f Kirkuk University, College of Science, Department of Chemistry, Kirkuk, Iraq

**Keywords:** crystal structure, Schiff base ligand, hydrogen bonding

## Abstract

In the title compound, C_18_H_18_N_2_O_4_, the planes of the benzene rings are twisted with respect to each other at 27.25 (7)°. The mol­ecule displays an extended conformation with an intra­molecular O—H⋯N hydrogen bond. In the crystal, weak C—H⋯O inter­actions link the mol­ecules, forming supra­molecular chains running along the *b-*axis direction.

## Related literature   

For a similar structure, see: Mague *et al.* (2015 ▸). For background to related Schiff base ligands and their biological activity, see: Adsule *et al.* (2006 ▸); Karthikeyan *et al.* (2006 ▸); Amimoto & Kawato (2005 ▸); Cohen & Schmidt (1964 ▸).
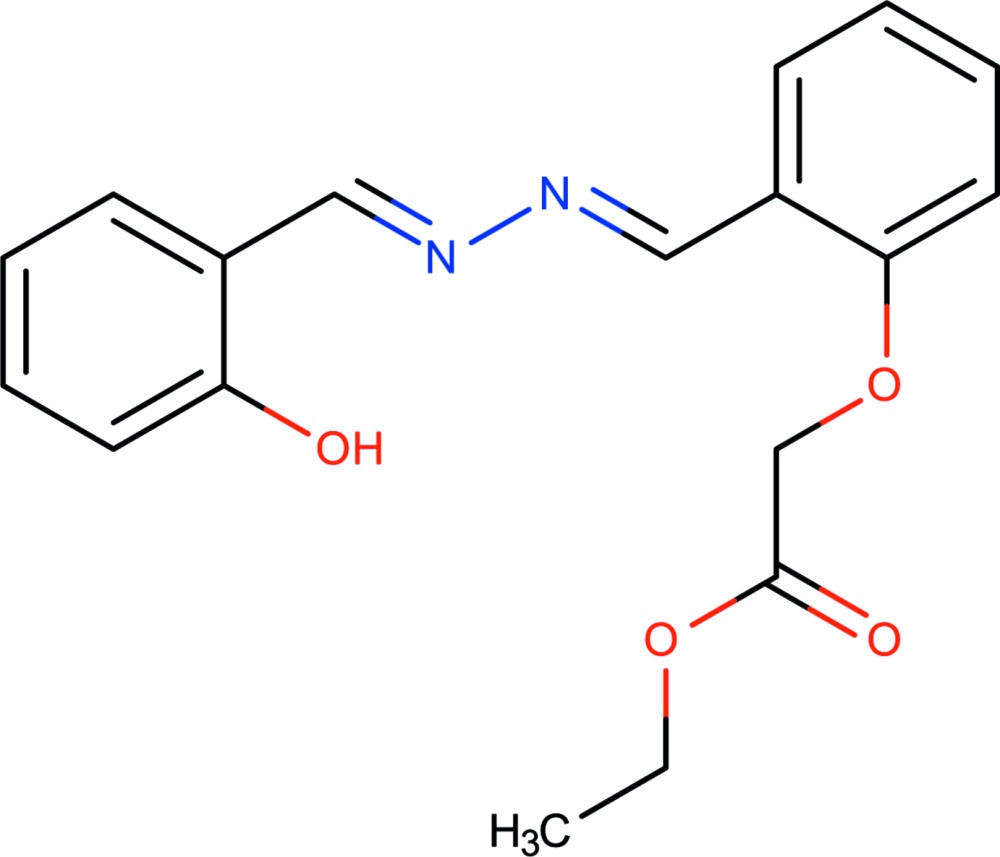


## Experimental   

### Crystal data  

C_18_H_18_N_2_O_4_
*M* *_r_* = 326.34
Monoclinic, 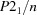

*a* = 17.6846 (4) Å
*b* = 4.8645 (1) Å
*c* = 19.2235 (4) Å
β = 107.357 (1)°
*V* = 1578.43 (6) Å^3^
*Z* = 4
Cu *K*α radiation
μ = 0.81 mm^−1^
*T* = 150 K
0.20 × 0.09 × 0.06 mm

### Data collection  

Bruker D8 VENTURE PHOTON 100 CMOS diffractometer
Absorption correction: multi-scan (*SADABS*; Bruker, 2014 ▸) *T*_min_ = 0.90, *T*_max_ = 0.95
11331 measured reflections
3063 independent reflections
2538 reflections with *I* > 2σ(*I*)
*R*_int_ = 0.031

### Refinement  

*R*[*F* ^2^ > 2σ(*F* ^2^)] = 0.038
*wR*(*F* ^2^) = 0.109
*S* = 1.06
3063 reflections
218 parameters
H-atom parameters constrained
Δρ_max_ = 0.24 e Å^−3^
Δρ_min_ = −0.21 e Å^−3^

### 

Data collection: *APEX2* (Bruker, 2014 ▸); cell refinement: *SAINT* (Bruker, 2014 ▸); data reduction: *SAINT*; program(s) used to solve structure: *SHELXT* (Sheldrick, 2008 ▸); program(s) used to refine structure: *SHELXL2014* (Sheldrick, 2008 ▸); molecular graphics: *DIAMOND* (Brandenburg & Putz, 2012 ▸); software used to prepare material for publication: *SHELXTL* (Sheldrick, 2008 ▸).

## Supplementary Material

Crystal structure: contains datablock(s) global, I. DOI: 10.1107/S2056989014027273/xu5832sup1.cif

Structure factors: contains datablock(s) I. DOI: 10.1107/S2056989014027273/xu5832Isup2.hkl

Supporting information file. DOI: 10.1107/S2056989014027273/xu5832Isup3.cml

**Fig. 1.**
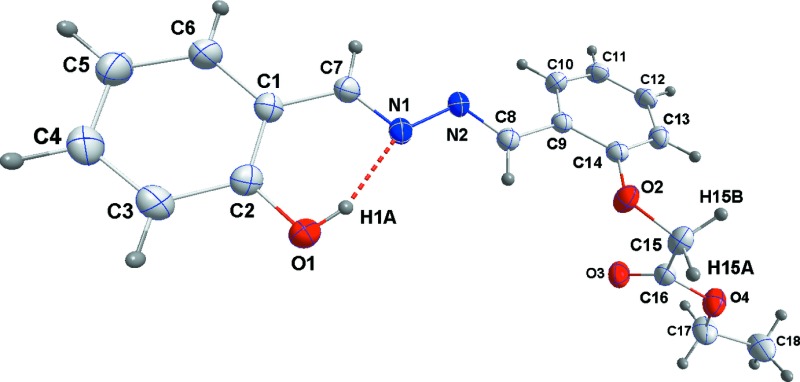
Perspective view of the title molecule with 50% probability ellipsoids and showing the atom labeling scheme and the intramolecular O—H···N hydrogen bond.

**Fig. 2.**
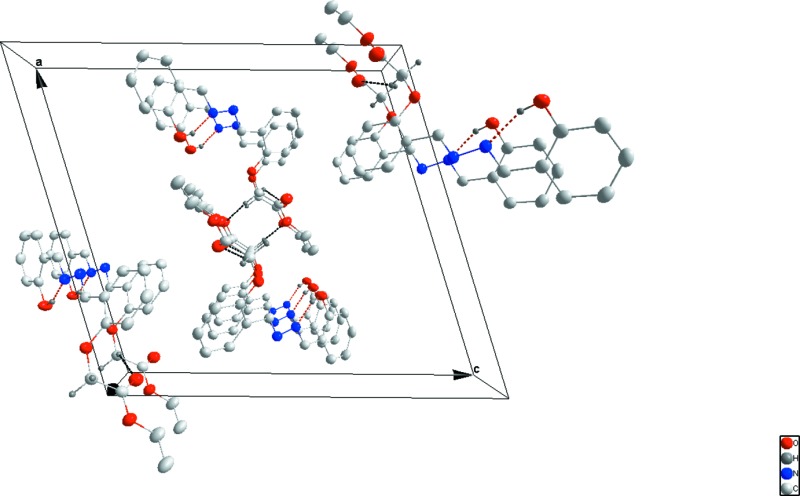
Packing viewed down the b axis showing C—H···O interactions as black dotted lines.

. DOI: 10.1107/S2056989014027273/xu5832fig1.tif
Perspective view of the title mol­ecule with 50% probability ellipsoids and showing the atom labeling scheme and the intra­molecular O—H⋯N hydrogen bond.

b . DOI: 10.1107/S2056989014027273/xu5832fig2.tif
Packing viewed down the *b* axis showing C—H⋯O inter­actions as black dotted lines.

CCDC reference: 1039095

Additional supporting information:  crystallographic information; 3D view; checkCIF report

## Figures and Tables

**Table 1:** Hydrogen-bond geometry (, )

*D*H*A*	*D*H	H*A*	*D* *A*	*D*H*A*
O1H1*A*N1	0.84	1.85	2.6441(17)	158
C15H15*A*O4^i^	0.99	2.58	3.3568(19)	136
C15H15*B*O3^ii^	0.99	2.57	3.440(2)	147

Symmetry codes: (i) 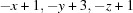; (ii) 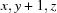.

## supplementary crystallographic information

### S1. Comment

Schiff bases of salicylaldehyde have gained importance from physiological and pharmacological activities point of view (Adsule *et al.*, 2006; Karthikeyan *et al.*, 2006). They also may exhibit thermochromism or photochromism depending on the planarity or nonplanarity, respectively, of the molecule (Amimoto & Kawato, 2005; Cohen & Schmidt, 1964). As part of our research efforts in the area of schiff base ligands we report in this study the synthesis and crystal structure determination of the title compound.

The title molecule is in an extended conformation with the phenyl rings C1–C6 and C9–C14, respectively, making dihedral angles of 7.4 (1)° and 19.8 (1)° with the mean plane of the central C7, N1, N2, C8 unit. The bond lengths and bond angles of the title molecule are normal and are comparable to those reported for a similar structure (Mague *et al.*, 2015).

The former angle is smaller as a result of the intramolecular O1—H1a···N1 hydrogen bond (Table 1). The packing consists of chains of molecules formed by weak C15—H15B···O3 interactions running parallel to the *b* axis with adjacent pairs of chains associated *via* C15—H15*a*···O4 interactions across centers of symmetry (Fig. 2 and Table 1).

### S2. Experimental

A mixture of 0.01 mol of 2-hydroxybenzohydrazide and 0.01 mol of ethyl 2-(2-formylphenoxy)acetate in 20 ml of ethanol was heated under reflux for 2 h. The solid product which precipitated from the hot solution was collected by filtration and dried under vacuum. Colourless crystals sufficient for X-ray diffraction were obtained by recrystallization from an ethanol solution. m.p. 428 K, yield 92%.

### S3. Refinement

H-atoms attached to carbon were placed in calculated positions (C—H = 0.95 - 0.99 Å) and refined in riding mode while hydroxyl-O atom was located in a difference Fourier map and refined by riding in its as-found relative position to oxygen atom. U_iso_(H) = 1.5U_eq_(C) for methyl H toms and 1.2U_eq_(C,O) for the others.

### Crystal data

**Table:**

C_18_H_18_N_2_O_4_	*F*(000) = 688
*M**_r_* = 326.34	*D*_x_ = 1.373 Mg m^−^^3^
Monoclinic, *P*2_1_/*n*	Cu *K*α radiation, λ = 1.54178 Å
*a* = 17.6846 (4) Å	Cell parameters from 6837 reflections
*b* = 4.8645 (1) Å	θ = 4.1–72.5°
*c* = 19.2235 (4) Å	µ = 0.81 mm^−^^1^
β = 107.357 (1)°	*T* = 150 K
*V* = 1578.43 (6) Å^3^	Column, colourless
*Z* = 4	0.20 × 0.09 × 0.06 mm

### Data collection

**Table:**

Bruker D8 VENTURE PHOTON 100 CMOS diffractometer	3063 independent reflections
Radiation source: INCOATEC IµS micro–focus source	2538 reflections with *I* > 2σ(*I*)
Mirror monochromator	*R*_int_ = 0.031
Detector resolution: 10.4167 pixels mm^-1^	θ_max_ = 72.5°, θ_min_ = 3.0°
ω scans	*h* = −18→21
Absorption correction: multi-scan (*SADABS*; Bruker, 2014)	*k* = −5→6
*T*_min_ = 0.90, *T*_max_ = 0.95	*l* = −23→23
11331 measured reflections	

### Refinement

**Table:**

Refinement on *F*^2^	Primary atom site location: structure-invariant direct methods
Least-squares matrix: full	Secondary atom site location: difference Fourier map
*R*[*F*^2^ > 2σ(*F*^2^)] = 0.038	Hydrogen site location: inferred from neighbouring sites
*wR*(*F*^2^) = 0.109	H-atom parameters constrained
*S* = 1.06	*w* = 1/[σ^2^(*F*_o_^2^) + (0.0535*P*)^2^ + 0.5415*P*] where *P* = (*F*_o_^2^ + 2*F*_c_^2^)/3
3063 reflections	(Δ/σ)_max_ < 0.001
218 parameters	Δρ_max_ = 0.24 e Å^−^^3^
0 restraints	Δρ_min_ = −0.21 e Å^−^^3^

### Special details

**Table:**

Geometry. All e.s.d.'s (except the e.s.d. in the dihedral angle between two l.s. planes) are estimated using the full covariance matrix. The cell e.s.d.'s are taken into account individually in the estimation of e.s.d.'s in distances, angles and torsion angles; correlations between e.s.d.'s in cell parameters are only used when they are defined by crystal symmetry. An approximate (isotropic) treatment of cell e.s.d.'s is used for estimating e.s.d.'s involving l.s. planes.
Refinement. Refinement of *F*^2^ against ALL reflections. The weighted *R*-factor *wR* and goodness of fit *S* are based on *F*^2^, conventional *R*-factors *R* are based on *F*, with *F* set to zero for negative *F*^2^. The threshold expression of *F*^2^ > σ(*F*^2^) is used only for calculating *R*-factors(gt) *etc*. and is not relevant to the choice of reflections for refinement. *R*-factors based on *F*^2^ are statistically about twice as large as those based on *F*, and *R*- factors based on ALL data will be even larger. H-atoms attached to carbon were placed in calculated positions (C—H = 0.95 - 0.99 Å) while that attached to oxygen was placed in a location derived from a difference map and its parameters adjusted to give O—H = 0.84 Å. All were included as riding contributions with isotropic displacement parameters 1.2 - 1.5 times those of the attached atoms.

### Fractional atomic coordinates and isotropic or equivalent isotropic displacement parameters (Å^2^)

**Table:**

	*x*	*y*	*z*	*U*_iso_*/*U*_eq_	
O1	0.26107 (6)	0.1064 (3)	0.62511 (6)	0.0392 (3)	
H1A	0.2450	0.2256	0.5924	0.047*	
O2	0.33072 (6)	1.1408 (2)	0.46429 (6)	0.0318 (3)	
O3	0.42576 (7)	0.9110 (2)	0.38921 (7)	0.0394 (3)	
O4	0.49863 (7)	1.2963 (2)	0.40435 (6)	0.0365 (3)	
N1	0.17349 (7)	0.4391 (3)	0.52416 (6)	0.0274 (3)	
N2	0.15351 (7)	0.6296 (3)	0.46651 (7)	0.0286 (3)	
C1	0.11819 (9)	0.1146 (3)	0.58929 (8)	0.0259 (3)	
C2	0.19168 (9)	0.0129 (3)	0.63240 (8)	0.0277 (3)	
C3	0.19421 (10)	−0.1914 (3)	0.68369 (8)	0.0322 (3)	
H3	0.2438	−0.2593	0.7130	0.039*	
C4	0.12504 (10)	−0.2958 (3)	0.69212 (8)	0.0333 (4)	
H4	0.1274	−0.4362	0.7271	0.040*	
C5	0.05185 (10)	−0.1982 (4)	0.65017 (9)	0.0355 (4)	
H5	0.0044	−0.2718	0.6561	0.043*	
C6	0.04881 (9)	0.0071 (3)	0.59976 (9)	0.0326 (4)	
H6	−0.0012	0.0767	0.5717	0.039*	
C7	0.11221 (9)	0.3247 (3)	0.53449 (8)	0.0273 (3)	
H7	0.0611	0.3800	0.5051	0.033*	
C8	0.21343 (9)	0.7639 (3)	0.46080 (8)	0.0259 (3)	
H8	0.2637	0.7370	0.4958	0.031*	
C9	0.20573 (8)	0.9589 (3)	0.40100 (8)	0.0248 (3)	
C10	0.13833 (9)	0.9571 (3)	0.33998 (8)	0.0298 (3)	
H10	0.0963	0.8334	0.3385	0.036*	
C11	0.13200 (9)	1.1324 (3)	0.28202 (8)	0.0323 (4)	
H11	0.0860	1.1286	0.2409	0.039*	
C12	0.19294 (9)	1.3133 (3)	0.28420 (8)	0.0314 (3)	
H12	0.1885	1.4344	0.2444	0.038*	
C13	0.26054 (9)	1.3203 (3)	0.34376 (8)	0.0291 (3)	
H13	0.3022	1.4450	0.3447	0.035*	
C14	0.26682 (8)	1.1433 (3)	0.40201 (8)	0.0251 (3)	
C15	0.39957 (9)	1.2843 (3)	0.46170 (8)	0.0312 (3)	
H15A	0.4364	1.3016	0.5117	0.037*	
H15B	0.3846	1.4718	0.4425	0.037*	
C16	0.44124 (9)	1.1381 (3)	0.41393 (8)	0.0290 (3)	
C17	0.54618 (11)	1.1827 (4)	0.36101 (11)	0.0423 (4)	
H17A	0.5114	1.1105	0.3142	0.051*	
H17B	0.5796	1.0304	0.3876	0.051*	
C18	0.59662 (11)	1.4085 (4)	0.34751 (11)	0.0444 (4)	
H18A	0.5629	1.5554	0.3199	0.067*	
H18B	0.6306	1.3375	0.3196	0.067*	
H18C	0.6297	1.4814	0.3942	0.067*	

### Atomic displacement parameters (Å^2^)

**Table:**

	*U*^11^	*U*^22^	*U*^33^	*U*^12^	*U*^13^	*U*^23^
O1	0.0279 (6)	0.0439 (7)	0.0432 (7)	−0.0035 (5)	0.0066 (5)	0.0125 (5)
O2	0.0294 (5)	0.0372 (6)	0.0291 (5)	−0.0080 (5)	0.0091 (4)	0.0026 (5)
O3	0.0414 (7)	0.0279 (6)	0.0506 (7)	−0.0041 (5)	0.0164 (5)	−0.0039 (5)
O4	0.0364 (6)	0.0330 (6)	0.0461 (7)	−0.0077 (5)	0.0212 (5)	−0.0072 (5)
N1	0.0294 (6)	0.0258 (7)	0.0276 (6)	−0.0011 (5)	0.0092 (5)	0.0035 (5)
N2	0.0296 (6)	0.0270 (7)	0.0299 (6)	0.0001 (5)	0.0101 (5)	0.0053 (5)
C1	0.0290 (7)	0.0236 (7)	0.0256 (7)	−0.0023 (6)	0.0091 (6)	−0.0019 (6)
C2	0.0291 (7)	0.0262 (8)	0.0270 (7)	−0.0039 (6)	0.0073 (6)	−0.0035 (6)
C3	0.0351 (8)	0.0310 (8)	0.0280 (7)	0.0007 (7)	0.0058 (6)	0.0022 (7)
C4	0.0459 (9)	0.0284 (8)	0.0286 (7)	0.0006 (7)	0.0157 (7)	0.0035 (6)
C5	0.0360 (8)	0.0355 (9)	0.0405 (9)	−0.0024 (7)	0.0197 (7)	0.0038 (7)
C6	0.0288 (8)	0.0336 (9)	0.0367 (8)	0.0000 (7)	0.0119 (6)	0.0036 (7)
C7	0.0259 (7)	0.0260 (8)	0.0301 (7)	0.0004 (6)	0.0084 (6)	0.0008 (6)
C8	0.0269 (7)	0.0243 (8)	0.0278 (7)	0.0006 (6)	0.0102 (6)	0.0002 (6)
C9	0.0266 (7)	0.0222 (7)	0.0280 (7)	0.0029 (6)	0.0121 (6)	0.0006 (6)
C10	0.0265 (7)	0.0300 (8)	0.0340 (8)	−0.0003 (6)	0.0108 (6)	0.0013 (7)
C11	0.0292 (8)	0.0357 (9)	0.0308 (8)	0.0051 (7)	0.0069 (6)	0.0045 (7)
C12	0.0372 (8)	0.0286 (8)	0.0314 (8)	0.0082 (7)	0.0147 (6)	0.0080 (7)
C13	0.0333 (8)	0.0251 (8)	0.0335 (8)	−0.0007 (6)	0.0170 (6)	0.0015 (6)
C14	0.0261 (7)	0.0244 (7)	0.0272 (7)	0.0025 (6)	0.0115 (6)	−0.0007 (6)
C15	0.0295 (8)	0.0331 (9)	0.0309 (8)	−0.0079 (7)	0.0090 (6)	−0.0026 (7)
C16	0.0275 (7)	0.0271 (8)	0.0302 (7)	−0.0022 (6)	0.0053 (6)	0.0018 (6)
C17	0.0435 (10)	0.0358 (10)	0.0562 (11)	0.0005 (8)	0.0280 (8)	−0.0050 (8)
C18	0.0440 (10)	0.0438 (11)	0.0537 (11)	0.0029 (8)	0.0273 (8)	0.0046 (9)

### Geometric parameters (Å, º)

**Table:**

O1—C2	1.3548 (18)	C8—C9	1.465 (2)
O1—H1A	0.8403	C8—H8	0.9500
O2—C14	1.3797 (17)	C9—C14	1.400 (2)
O2—C15	1.4172 (17)	C9—C10	1.401 (2)
O3—C16	1.2016 (19)	C10—C11	1.381 (2)
O4—C16	1.3295 (18)	C10—H10	0.9500
O4—C17	1.4577 (19)	C11—C12	1.382 (2)
N1—C7	1.2854 (19)	C11—H11	0.9500
N1—N2	1.4064 (17)	C12—C13	1.387 (2)
N2—C8	1.2772 (19)	C12—H12	0.9500
C1—C6	1.402 (2)	C13—C14	1.390 (2)
C1—C2	1.406 (2)	C13—H13	0.9500
C1—C7	1.449 (2)	C15—C16	1.515 (2)
C2—C3	1.391 (2)	C15—H15A	0.9900
C3—C4	1.378 (2)	C15—H15B	0.9900
C3—H3	0.9500	C17—C18	1.486 (3)
C4—C5	1.389 (2)	C17—H17A	0.9900
C4—H4	0.9500	C17—H17B	0.9900
C5—C6	1.381 (2)	C18—H18A	0.9800
C5—H5	0.9500	C18—H18B	0.9800
C6—H6	0.9500	C18—H18C	0.9800
C7—H7	0.9500		
			
C2—O1—H1A	101.2	C9—C10—H10	119.5
C14—O2—C15	117.06 (11)	C10—C11—C12	119.60 (14)
C16—O4—C17	117.01 (13)	C10—C11—H11	120.2
C7—N1—N2	112.44 (12)	C12—C11—H11	120.2
C8—N2—N1	112.64 (12)	C11—C12—C13	120.84 (14)
C6—C1—C2	118.59 (14)	C11—C12—H12	119.6
C6—C1—C7	119.35 (14)	C13—C12—H12	119.6
C2—C1—C7	122.06 (13)	C12—C13—C14	119.45 (14)
O1—C2—C3	118.34 (14)	C12—C13—H13	120.3
O1—C2—C1	121.82 (14)	C14—C13—H13	120.3
C3—C2—C1	119.84 (14)	O2—C14—C13	123.53 (13)
C4—C3—C2	120.30 (15)	O2—C14—C9	115.80 (12)
C4—C3—H3	119.9	C13—C14—C9	120.65 (14)
C2—C3—H3	119.9	O2—C15—C16	111.61 (13)
C3—C4—C5	120.80 (15)	O2—C15—H15A	109.3
C3—C4—H4	119.6	C16—C15—H15A	109.3
C5—C4—H4	119.6	O2—C15—H15B	109.3
C6—C5—C4	119.29 (15)	C16—C15—H15B	109.3
C6—C5—H5	120.4	H15A—C15—H15B	108.0
C4—C5—H5	120.4	O3—C16—O4	124.80 (15)
C5—C6—C1	121.17 (15)	O3—C16—C15	125.22 (14)
C5—C6—H6	119.4	O4—C16—C15	109.98 (13)
C1—C6—H6	119.4	O4—C17—C18	107.37 (14)
N1—C7—C1	122.39 (14)	O4—C17—H17A	110.2
N1—C7—H7	118.8	C18—C17—H17A	110.2
C1—C7—H7	118.8	O4—C17—H17B	110.2
N2—C8—C9	120.88 (13)	C18—C17—H17B	110.2
N2—C8—H8	119.6	H17A—C17—H17B	108.5
C9—C8—H8	119.6	C17—C18—H18A	109.5
C14—C9—C10	118.41 (13)	C17—C18—H18B	109.5
C14—C9—C8	120.88 (13)	H18A—C18—H18B	109.5
C10—C9—C8	120.65 (13)	C17—C18—H18C	109.5
C11—C10—C9	121.06 (14)	H18A—C18—H18C	109.5
C11—C10—H10	119.5	H18B—C18—H18C	109.5
			
C7—N1—N2—C8	173.32 (13)	C8—C9—C10—C11	−177.30 (14)
C6—C1—C2—O1	179.77 (14)	C9—C10—C11—C12	−0.2 (2)
C7—C1—C2—O1	0.1 (2)	C10—C11—C12—C13	0.3 (2)
C6—C1—C2—C3	0.4 (2)	C11—C12—C13—C14	−0.2 (2)
C7—C1—C2—C3	−179.22 (14)	C15—O2—C14—C13	−13.6 (2)
O1—C2—C3—C4	−178.94 (14)	C15—O2—C14—C9	168.16 (13)
C1—C2—C3—C4	0.4 (2)	C12—C13—C14—O2	−178.18 (13)
C2—C3—C4—C5	−0.5 (2)	C12—C13—C14—C9	0.0 (2)
C3—C4—C5—C6	−0.4 (3)	C10—C9—C14—O2	178.35 (13)
C4—C5—C6—C1	1.2 (3)	C8—C9—C14—O2	−4.28 (19)
C2—C1—C6—C5	−1.3 (2)	C10—C9—C14—C13	0.0 (2)
C7—C1—C6—C5	178.39 (15)	C8—C9—C14—C13	177.39 (13)
N2—N1—C7—C1	177.86 (13)	C14—O2—C15—C16	−71.06 (17)
C6—C1—C7—N1	177.23 (14)	C17—O4—C16—O3	−0.4 (2)
C2—C1—C7—N1	−3.1 (2)	C17—O4—C16—C15	178.44 (14)
N1—N2—C8—C9	175.83 (12)	O2—C15—C16—O3	−9.9 (2)
N2—C8—C9—C14	167.75 (14)	O2—C15—C16—O4	171.21 (12)
N2—C8—C9—C10	−14.9 (2)	C16—O4—C17—C18	170.82 (14)
C14—C9—C10—C11	0.1 (2)		

### Hydrogen-bond geometry (Å, º)

**Table:**

*D*—H···*A*	*D*—H	H···*A*	*D*···*A*	*D*—H···*A*
O1—H1*A*···N1	0.84	1.85	2.6441 (17)	158
C15—H15*A*···O4^i^	0.99	2.58	3.3568 (19)	136
C15—H15*B*···O3^ii^	0.99	2.57	3.440 (2)	147

Symmetry codes: (i) −*x*+1, −*y*+3, −*z*+1; (ii) *x*, *y*+1, *z*.
