# Supplementary figures and images for: Crystal structure of ethyl 2-(2-{(1E)-[(E)-2-(2-hy­droxy­benzyl­idene)hydrazin-1-yl­idene]meth­yl}phen­oxy)acetate
Source: Acta Crystallogr E Crystallogr Commun. 2015 Jan 3;71(Pt 2):o70–1. doi: 10.1107/S2056989014027273 (PMC4384580; doi:10.1107/S2056989014027273)

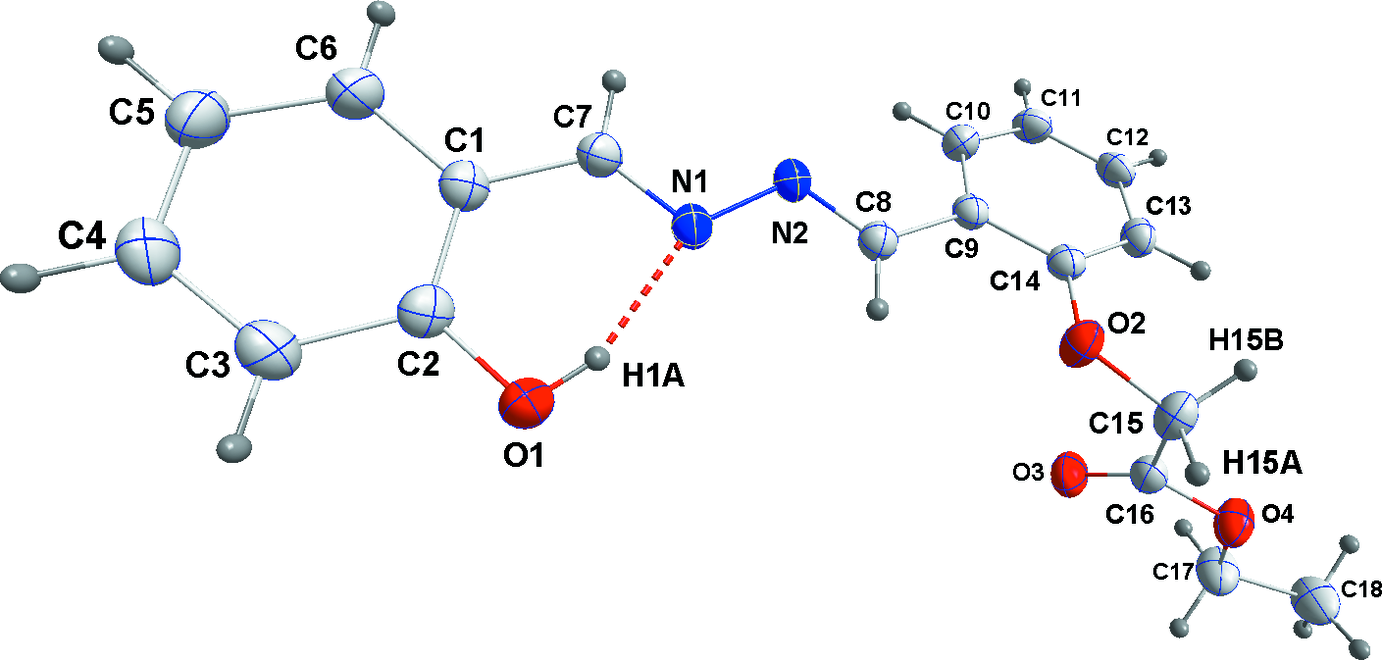

Supplement: Supplementary file 4 [file e-71-00o70-fig1.tif]

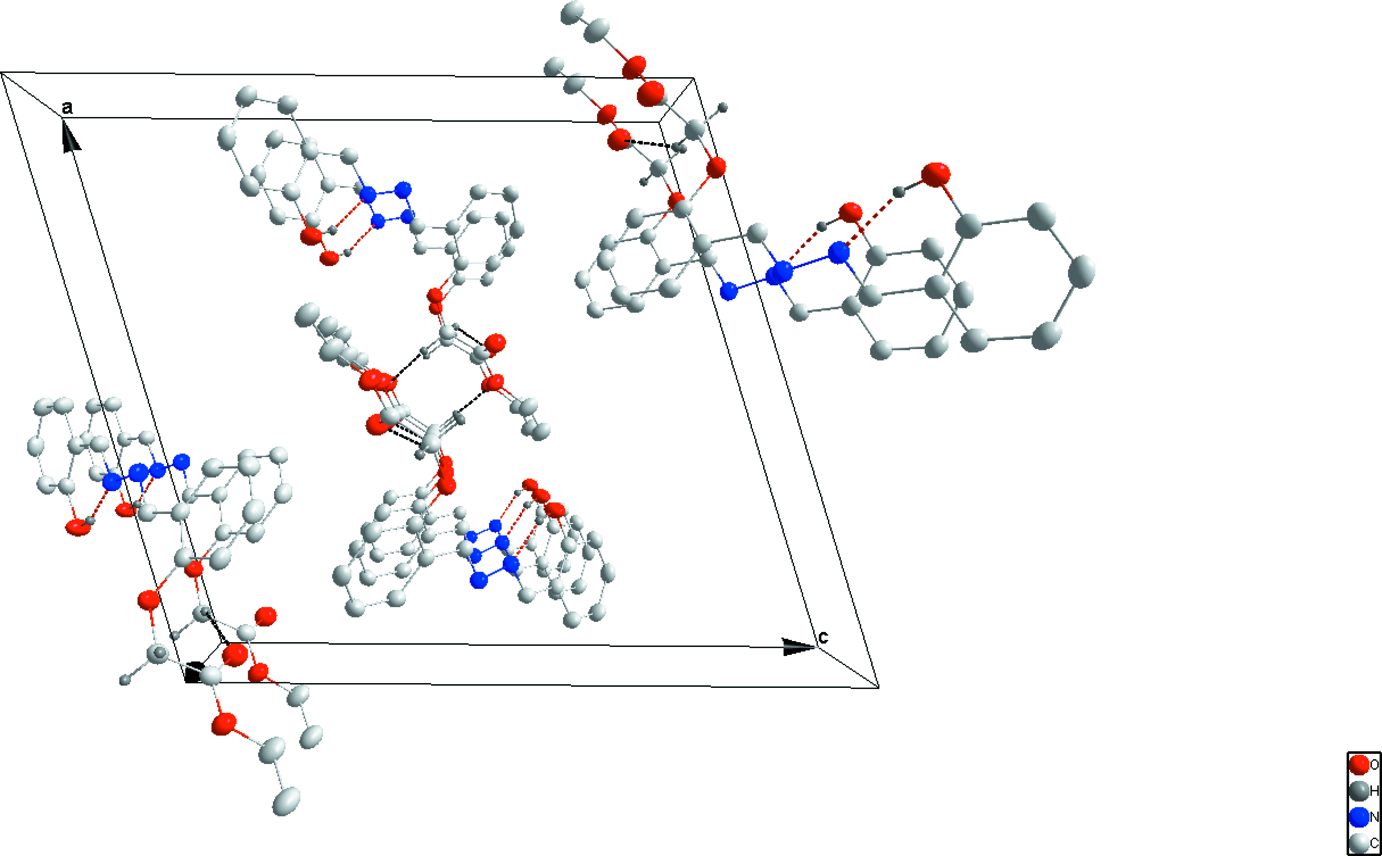

Supplement: Supplementary file 5 [file e-71-00o70-fig2.tif]
